# MicroRNAs as Predictive Biomarkers of Resistance to Targeted Therapies in Gastrointestinal Tumors

**DOI:** 10.3390/biomedicines9030318

**Published:** 2021-03-21

**Authors:** Valentina Angerilli, Francesca Galuppini, Gianluca Businello, Luca Dal Santo, Edoardo Savarino, Stefano Realdon, Vincenza Guzzardo, Lorenzo Nicolè, Vanni Lazzarin, Sara Lonardi, Fotios Loupakis, Matteo Fassan

**Affiliations:** 1 Surgical Pathology & Cytopathology Unit, Department of Medicine (DIMED), University of Padua, 35100 Padua, Italy; valentina.angerilli@gmail.com (V.A.); francesca.galuppini@unipd.it (F.G.); glc.businello@gmail.com (G.B.); lucas1186dalsanto@gmail.com (L.D.S.); vincenza.guzzardo@unipd.it (V.G.); lorenzo.nic86@gmail.com (L.N.); vanni.lazzarin@unipd.it (V.L.); 2 Division of Gastroenterology, Department of Surgical, Oncological and Gastroenterological Sciences, University of Padua, 35100 Padua, Italy; edoardo.savarino@unipd.it; 3 Istituto Oncologico Veneto (IOV-IRCCS), 35100 Padua, Italy; stefano.realdon@iov.veneto.it (S.R.); sara.lonardi@iov.veneto.it (S.L.); fotios.loupakis@iov.veneto.it (F.L.)

**Keywords:** microRNAs, GI cancers, targeted therapy, drug resistance

## Abstract

The advent of precision therapies against specific gene alterations characterizing different neoplasms is revolutionizing the oncology field, opening novel treatment scenarios. However, the onset of resistance mechanisms put in place by the tumor is increasingly emerging, making the use of these drugs ineffective over time. Therefore, the search for indicators that can monitor the development of resistance mechanisms and above all ways to overcome it, is increasingly important. In this scenario, microRNAs are ideal candidate biomarkers, being crucial post-transcriptional regulators of gene expression with a well-known role in mediating mechanisms of drug resistance. Moreover, as microRNAs are stable molecules, easily detectable in tissues and biofluids, they are the ideal candidate biomarker to identify patients with primary resistance to a specific targeted therapy and those who have developed acquired resistance. The aim of this review is to summarize the major studies that have investigated the role of microRNAs as mediators of resistance to targeted therapies currently in use in gastro-intestinal neoplasms, namely anti-EGFR, anti-HER2 and anti-VEGF antibodies, small-molecule tyrosine kinase inhibitors and immune checkpoint inhibitors. For every microRNA and microRNA signature analyzed, the putative mechanisms underlying drug resistance were outlined and the potential to be translated in clinical practice was evaluated.

## 1. Introduction

The number of druggable tumor-specific molecular alterations has grown substantially in the past decade and a great survival benefit has been obtained from genomic-driven therapies across many cancer types. However, while molecularly targeted drugs offer a significantly higher response rate than traditional chemotherapy in diseases such as melanoma and non-small cell lung cancer, they have shown relatively modest clinical benefits in gastrointestinal (GI) malignancies [1]. The “one gene, one drug” approach of precision oncology clashes with the reality of the extremely complex molecular landscape of most solid tumors. A crucial aspect towards a successful development and application of targeted therapies is the understanding of resistance mechanisms that limit their effectiveness [1].

Primary or intrinsic resistance is defined as radiographic or clinical disease progression as the best response to an anticancer therapy and implies the pre-existence of resistance-mediating factors within the tumor [2].

Secondary or acquired resistance is defined as therapeutic resistance after an initial period of disease stabilization or response, and be can caused by mutations arising during treatment, as well as through various adaptative mechanisms [3].

A wide range of biological determinants have been associated with drug resistance to targeted therapies; these include the presence of undruggable genomic drivers, the mutation of drug targets, the activation of survival signaling pathways, and the inactivation of downstream death signaling pathways. The tumor microenvironment may also mediate resistance by numerous mechanisms, including: promoting immune evasion of cancer cells, hampering drug absorption and stimulating paracrine cancer cell growth factors. The epithelial–mesenchymal transition (EMT) process and the presence of tumor stem cells have also been identified as causes of drug resistance. Furthermore, the coexistence of genetically and molecularly heterogeneous subclones within the bulk solid tumor may substantially fuel resistance under therapeutic selective pressure [2,4,5].

The purpose of this review is to elucidate the role of microRNAs (miRNAs) as mediators of resistance to targeted therapies in GI tumors.

## 2. MicroRNAs and Drug Resistance

MiRNAs are a group of short non-coding RNA transcripts (18–22 nucleotides) that control gene expression at the post-transcriptional level, thus regulating several pathways involved in the maintenance of cell homeostasis. In most cases, they act by binding to the 3′-untranslated region (3′-UTR) of their target messenger RNAs (mRNAs), which results in mRNA silencing or degradation and subsequent post-translational degradation or down-modulation of proteins [6,7].

**Figure 1:**
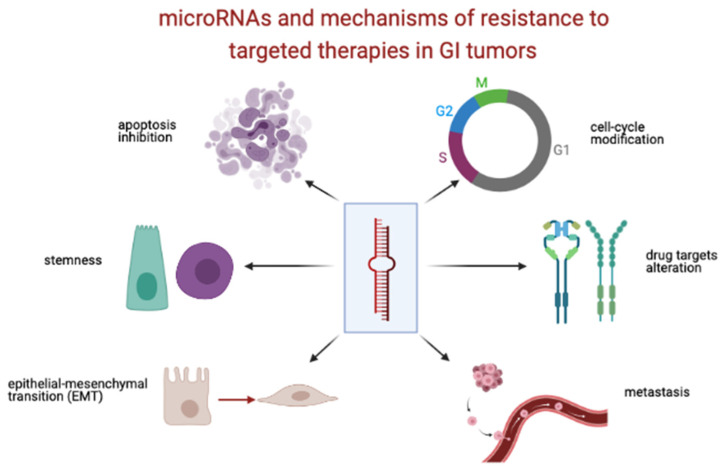
MiRNAs and mechanisms of resistance to targeted therapies in GI tumors. Credits to BioRender.com, accessed date 18 March 2021.

Numerous studies have demonstrated that miRNAs are heavily dysregulated in cancer and in certain conditions they may function as oncogenes or tumor suppressor genes. Hence, microRNAs have been identified as potential diagnostic, prognostic and predictive biomarkers for patient stratification in oncology [8,9]. Particularly, miRNAs have been found to act as mediators of drug resistance through various cellular and molecular mechanisms, which are related to: apoptosis, cell cycle modification, alteration in drug targets, regulation of drug efflux transporters, epithelial-mesenchymal transition (EMT) and cancer stem cells [10,11,12] (Figure 1).

In light of their high stability in tissues and bodily fluids (i.e., plasma and urine), miRNAs are ideal candidates to monitor cancer treatment resistance. In fact, miRNAs are not only present within cells, but are also actively secreted from cells, and included in RNA-binding multiprotein complexes and/or exosomes [13]. In the context of liquid biopsies, analysis of miRNA expression patterns represents a promising tool to perform non-invasive longitudinal tracking of drug sensitivity over time [14,15].

When referring to GI malignancies, the role of miRNAs as predictors of resistance to chemotherapy has been extensively studied [16,17,18], while further research is needed to gain a better understanding of the complex interplay between miRNA expression and resistance to targeted therapy. Furthermore, many studies have documented the change in miRNA expression profiles following radiotherapy, so that miRNAs could prove useful in modulating radioresponsitivity at the clinical level [19].

With regard to future perspectives, playing such an important role in carcinogenesis, miRNAs have also been proposed and tested as a therapeutic option. In cancer, in order to increase the level of a tumor suppressor miRNA, whose level is downregulated, miRNA mimics can be used to re-establish normal expression and function of a certain miRNA of interest. On the other hand, antagomiRs can be used to suppress the function of a specific oncomiRNA that play a role in the development and progression of the neoplasm. However, while many studies have attempted to evaluate the consequences of the manipulation of these molecules with the purpose to support findings from in vitro or in vivo models, only a few miRNA targeted drugs have entered clinical trials because of a few important limitations, such as dosage and cross-reactions with other miRNAs [20].

## 3. Anti-EGFR

Epidermal growth factor receptors (EGFRs) are a large family of receptor tyrosine kinases expressed in several types of cancer and are responsible for the activation of a downstream signaling cascade that modulates growth, signaling, differentiation, adhesion, migration and survival of cancer cells [21].

Targeting EGFR has been revealed to be a successful strategy in metastatic colorectal cancer (mCRC). Cetuximab and panitumumab are the two anti-EGFR antibodies currently in use for mCRC [22]. Anti-EGFR antibodies act by binding the extra-cellular domain of EGFR, thereby preventing ligand binding and blocking the downstream signaling pathway. Furthermore, they induce receptor internalization and degradation [23], and are responsible for antibody-dependent cellular cytotoxicity [24].

In patients with metastatic disease, anti-EFGR monoclonal antibodies have shown a clinically significant response when given in monotherapy or in combination with chemotherapy [25,26]. However, drug resistance frequently arises. Many genomic and non-genomic mechanisms underlie resistance to anti-EGFR therapy. Particularly, a plethora of overlapping de novo and acquired genetic alterations have been identified, such as *KRAS*, *NRAS*, *BRAF*, *PIK3C,* and *EFGR* mutations and *MET* and *ERBB2* amplifications [27,28].

*Let-7. KRAS* mutations are the major predictors of resistance to EGFR inhibitors and are routinely screened in mCRCs [29]. In this regard, members of the Lethal-7 (Let-7) family of miRNAs have been found to display KRAS downregulating activity by binding to specific sites in the 3′-UTR of KRAS mRNA [30]. Ruzzo et al. measured the expression of let-7 in formalin-fixed paraffin-embedded (FFPE) tumor samples of mCRC patients who underwent third-line therapy with cetuximab plus irinotecan. The findings of this study revealed that higher levels of let-7 were significantly associated with better survival outcome in patients, regardless of *KRAS* mutational status. In patients with *KRAS* mutations, let-7 may serve to identify a subpopulation of responders to anti-EGFR therapy [31]. Furthermore, Cappuzzo et al. found that the high-intensity signature of the cluster Let-7c/miR-99a/miR-125b is associated with a favorable response to cetuximab and panitumumab in *KRAS* wild-type mCRC patients [32].

*MiR-31-5p/miR-31-3p.* The predictive role of miR-31-5p and miR-31-3p has been investigated in several studies. In a study which included an exploratory cohort and a validation cohort, of the 9 miRNAs analyzed in FFPE tumor samples of both cetuximab responders and non-responders, miRNA-31-3p and miRNA-31-5p were found to be strongly associated with time to progress (TTP) in wild-type *RAS* patients treated with cetuximab [33]. In another study it was further discovered that high miR-31-5p expression was associated with shorter progression-free survival (PFS) in mCRC patients treated with anti-EGFR therapy [34]. Furthermore, Manceau et al., after screening 1145 miRNAs on a large cohort of wild-type *RAS* mCRC patients, identified miR-31-3p as a promising predictive biomarker of response to anti-EGFR antibodies [35].

*MiR-10/miR-125b.* MiR-10 and miR-125b are generated by the long non-coding RNA (lncRNA) MIR100HG, and decrease the expression of five negative regulators of the Wnt/β-catenin pathways, resulting in increased Wnt signaling, which is a hallmark of colorectal carcinogenesis. It was demonstrated that the overexpression of lncRNA MIR100HG and the two embedded miRNAs was associated with cetuximab resistance in tumors derived from CRC patients and in vitro 3-D cell culture models [36].

*MiR-181a.* MiR-181a has a strong tumor-promoting effect via inhibition of the tumor suppressor factor Wnt Inhibitory factor 1 (WIF-1) and stimulates tumor growth, cell motility and invasion. Pichler et al. analyzed the expression of miR-181a in FFPE tumor samples of a cohort of patients with *KRAS* wild-type mCRC, undergoing cetuximab and panitumumab treatment. The findings of the study highlighted the potential predictive role of miR-181a, as high levels of this miRNA were associated with a longer PFS [37].

*MiR-345.* According to the results of a study by Schou and colleagues, high levels of miR-345 in whole blood correlated with a lack of response to therapy in patients treated with third-line cetuximab and irinotecan [38]. In the context of liquid biopsy, miR-345 appears to be a valid candidate biomarker of sensitivity to anti-EGFR therapy.

*MiR-199a/miR-375.* Mussnich et al. performed miRNA expression profiling of human CRC cell lines sensitive to cetuximab and of their resistant counterparts. Among the investigated miRNAs, miR-199a and miR-375 were found to be overexpressed in resistant cells and their upregulation promoted cetuximab resistance. MiR-199a and miR-375 both target PH Domain And Leucine Rich Repeat Protein Phosphatase 1 (PHLPP1), which acts as a tumor suppressor by downregulating the Akt oncogenic pathway [39].

*MiR-302.* In a recent study, downregulation of miR-302a was observed in cetuximab-resistant CRC cells as well as in patient-derived xenografts. Furthermore, miR-302a overexpression restored cetuximab responsiveness in CRC cell lines both in vitro and in vivo. MiR-302a has been found to act as a tumor suppressor by targeting NFIB (Nuclear factor 1 B-type) and downregulating the NFIB/ITGA6 axis, which is responsible for cell migration and invasion in the metastatic process [40].

*MiR-141-3p.* The findings of a study by Xing and colleagues indicated that miR-141-3p might be a predictor of response to cetuximab. In CRC cell cultures, miR-141-3p regulated cetuximab sensitivity by directly targeting EGFR and its downstream cascade. Moreover, miR-141-3p improved cetuximab-induced apoptosis in CRC cells, proving to have a tumor-suppressing function [41].

## 4. Anti-VEGF

Vascular endothelial growth factor (VEGF) promotes tumor angiogenesis through several mechanisms, including enhanced endothelial cell proliferation, survival and migration, chemotaxis of bone-marrow-derived progenitor cells, vascular permeability, and vasodilation. VEGF ligand acts by binding the VEGF receptor (VEGFR) with tyrosine kinase activity, which activates a network of downstream signaling pathways, responsible for mediating numerous changes within the tumor vasculature. Several strategies have been put in place to inhibit the VEGF pathway, including monoclonal antibodies blocking VEGF or VEGFR, soluble VEGF receptors, and tyrosine kinase inhibitors selective for VEGFRs [42].

Bevacizumab is a monoclonal antibody directed against VEGF and has been approved in combination with cytotoxic chemotherapy as first or second-line therapy in mCRC, after several randomized clinical trials had shown improvements in overall survival [43,44,45]. The VEGF decoy receptor aflibercept has also recently been introduced in clinical practice as second-line therapy in mCRC [46].

In the context of gastro-esophageal malignancies, the anti-VEGFR2 antibody ramucirumab is currently licensed for clinical use in combination with cytotoxic chemotherapy for chemo-refractory metastatic disease [47,48]. Sunitinib and sorafenib are multi-tyrosine kinase inhibitors (TKIs), which target a wide spectrum of pathways involved in tumor growth, including VEGF/VEGFR, and are in use in GI precision oncology.

However, the VEGF pathway inhibitors are failing to provide a durable response in most patients. The activation of alternative angiogenic “escape” pathways is a major contributor to drug resistance and may induce tumor growth, enhancement of invasion and metastasis [49]. A few preliminary studies have been carried out to elucidate the role of miRNAs as mediators of resistance to bevacizumab, however there is still no evidence regarding ramucirumab or aflibercept.

*MiR-126.* MiR-126 acts by enhancing the proangiogenic actions of VEGF and promotes blood vessel formation and survival [50]. MiR-126-containing micro-vesicles originate from endothelial cells and fuse with the membrane of neighboring cells, thus promoting angiogenesis in a paracrine manner. A negative relationship exists between levels of circulating miR-126 and their response to bevacizumab. Increasing plasma levels of miR-126 are associated with resistance to bevacizumab, while lower levels indicate treatment response. As miR-126 is representative of endothelial cell turn-over, its circulating levels could help perform non-invasive monitoring of bevacizumab resistance.

*MiR-664-3p/miR-455-5p*. The predictive role of miR-664-3p and miR-455-5p has been evaluated in a study by Boisen et al. Higher miR-664-3p and lower miR-455-5p expression evaluated in FFPE tumor samples was found to be associated with an improved outcome in the cohorts of patients treated with bevacizumab and chemotherapy, in comparison with the cohort treated with chemotherapy only. Therefore, miR-664-3p, with its putative tumor suppressor role, and miR-455-5p, with its putative oncogenic role, could represent potential predictive tissue biomarkers of bevacizumab effectiveness [51].

*MiR-20b-5p/miR-29b-3p/miR-155-5p.* According to the results of a study by Ulivi and colleagues, higher circulating levels of miR-20b-5p, miR-29b-3p and miR-155-5p are associated with a better outcome in mCRC patients treated with a combination of bevacizumab and chemotherapy. Furthermore, the variation in plasma levels of miR-155-5p could also be indicative of patient survival. In line with these findings, previous studies have found that miR-20b, miR-29b and miR-155-5p play a role in regulating tumor angiogenesis [52]. MiR-29b acts as a tumor suppressor through simultaneously inhibiting angiogenesis and tumorigenesis by targeting Akt3 [53]. Although little information is available on its involvement in the tumor angiogenic process, miR-20 has been found to regulate proliferation and senescence in endothelial cells [54]. MiR-155 contributes to controlling hypoxia-inducible factor (HIF-1α) and promotes angiogenesis under hypoxia condition [55].

## 5. Anti-HER2

HER2 belongs to the EGFR tyrosine kinase family. It is localized in the cell membrane and when activated initiates intracellular downstream signaling involved in diverse biological processes related to cancer, such as proliferation, migration and apoptosis [56].

Overexpression of HER2 has been detected in 11–20% of gastric and gastroesophageal junction (GEJ) cancers [57,58,59,60]. Trastuzumab is an anti-HER2 antibody that acts by blocking the activity of the HER2 receptor and weakening the downstream signaling [61].

In 2010, the results of the Trastuzumab for Gastric cancer (ToGA) trial displayed how a combination of trastuzumab and chemotherapy was able to prolong the overall survival compared to chemotherapy alone in gastric cancer patients with HER2 overexpression. Thus, trastuzumab in combination with chemotherapy has been approved as the standard first-line treatment of advanced gastric or GEJ cancer with HER2 overexpression/*ERBB2* amplification [62]. However, only a fraction of the patients has been found to respond to trastuzumab, and even those who achieved an initial therapeutic response developed resistance within 7 months [63].

*MiR-21.* It has been found that trastuzumab activates phosphatase and tensin homolog (PTEN) phosphatase, thus decreasing PTEN tyrosine phosphorylation, via inhibition of HER2 receptor bound Src. Reduced PTEN expression could predict trastuzumab resistance [64]. According to the results of a study by Eto et al., overexpression of miR-21 downregulates PTEN and increases the phosphorylation of PTEN downstream target Akt in HER2-positive gastric cancer (GC) cell lines, thus resulting in decreased sensitivity to trastuzumab-induced apoptosis. The opposite effect in PTEN and p-Akt was observed after miR-21 suppression, which instead restored resistance to trastuzumab. These findings suggest that the miR-21/PTEN pathway might play a critical role in regulating trastuzumab resistance of GC cells via modulating apoptosis [65].

*MiR-223.* F-box and WD repeat domain-containing 7 (FBXW7) is the substrate recognition component of an evolutionarily conserved ubiquitin ligase complex, which appears to have an important role in controlling the stability of several oncoprotein substrates, including cyclin E, c-Myc, Notch, c-Jun, mammalian target of rapamycin (mTOR) and Myeloid Cell Leukemia 1 (MCL1) [66]. Upregulation of miR-223 decreased FBXW7 expression and subsequently reduced the sensitivity of HER2-positive GC cell lines to trastuzumab, thereby acting as an oncomiR and suppressing trastuzumab-induced apoptosis. On the contrary, downregulation of miR-223 restored FBXW7 expression and the sensitivity to trastuzumab [67].

*MiR-16.* Trastuzumab has the ability to block PI3K/AKT downstream signaling, which results in the inhibition of c-Myc activation and subsequent upregulation of miR-16. A study by Venturutti et al. identified cyclin J and Far Upstream Element Binding Protein 1 (FUBP1) as miR-16 targets. miR-16 acts as a tumor suppressor by exerting an antiproliferative effect via silencing its miRNA targets [68]. Furthermore, it was found that in vitro overexpression of miR-16 and low or null levels of FUBP1 were predictors of trastuzumab sensitivity. These findings suggest that both miR-16 and FUBP1 could represent promising predictive biomarkers of response to trastuzumab [69].

*MiR-125b.* MiR-125b has been found to be dysregulated in several cancer types. For example, while being downregulated in osteosarcoma, breast cancer, ovarian cancer and hepatocellular carcinoma (HCC), it appears to be upregulated in CRC, prostate cancer, non-small cell lung cancer and GC [70]. In the latter, miR-125b functions as an oncogene by promoting cellular proliferation, migration and invasion by downregulating the expression of Protein Phosphatase 1 Catalytic Subunit Alpha (PPP1CA) and upregulating Rb phosphorylation. Furthermore, it has been recently demonstrated that high miR-125b levels are associated with poor prognosis in patients with HER2-positive GC treated with trastuzumab, thus indicating a speculative role of miR-125b in trastuzumab resistance [71].

*MiR-200c.* Compelling evidences identify in the EMT one of the mechanisms implicated in drug resistance [72]. In a recent study by Zhou et al. it was observed that trastuzumab-resistant GC cell cultures expressed high levels of EMT markers, as well as TGF-β, which is a master regulator of the EMT. Furthermore, while miR-200c was found to be downregulated in trastuzumab-resistant GC cells, thereby proving to act as a tumor suppressor miRNA, its overexpression restored trastuzumab sensitivity and blocked the EMT by targeting Zinc finger E-box-binding homeobox 1 (ZEB1) and 2 (ZEB2), which are downstream molecules of TGF-β. This study suggested that the TGF-β/ZEB/miR-200c axis is involved in the resistance of trastuzumab in GC by regulating the EMT [73].

*MiR-494.* Lapatinib is a small molecule which inhibits the tyrosine kinases of HER2 and EGFR1 [74]. MiR-494 has been found to act as a tumor suppressor and to restore lapatinib sensitivity and inhibit formation of cancer-initiating cells (CICs) via reducing expression of FGFR2 in HER2-positive, FGFR2 overexpressing and lapatinib resistant GC cell cultures [75].

## 6. Small-Molecule Tyrosine Kinase Inhibitors

Receptor tyrosine kinases (RTKs) are widely expressed transmembrane proteins that regulate many fundamental cellular processes, and play a key role in the physiopathology of several diseases. Upon ligand binding, RTKs activate intracellular signaling pathways involved in many functions, such as: differentiation, proliferation, migration, invasion and angiogenesis. Aberrant RTK expression being a well-recognized mechanism of tumorigenesis, a broad variety of inhibitors are currently in clinical use across many cancer types [76]. Particularly, small-molecule tyrosine kinase inhibitors act by blocking the intracellular domain of the receptor or by inhibiting the tyrosine kinase activity of downstream signaling mediators [77].

### 6.1. Imatinib

Imatinib is a selective inhibitor of certain tyrosine kinases and is highly active in patients with gastrointestinal stromal tumors (GISTs) by blocking the constitutive activity of KIT and platelet-derived growth factor receptor α (PDGFR-α). Imatinib is the treatment of choice for advanced GISTs harboring *KIT* and *PDGFR-α* mutations, and it is also used in adjuvant and neoadjuvant settings. Although imatinib has high response rates, drug resistance remains the main challenge for extending patient survival [78]. The mechanisms underlying imatinib resistance are not completely clear. However, in about half of patients, resistance is caused by secondary mutations in *KIT* (exons 13, 14, 17 or 18) [79].

*MiR-125a-5p.* In a study on FFPE GIST samples from patients resistant to imatinib in a neoadjuvant setting, it was found that miR-125a-5p can modulate imatinib response in *KIT*-mutated GIST cells by regulating the expression of Protein Tyrosine Phosphatase Non-Receptor Type 18 (PTPN18). Because the targets of miR-125a-5p have been functionally associated with anti-apoptosis, cell cycle progression, signal transduction and protein phosphorylation, miR-125a-5p acts as oncomiR and its overexpression was linked with imatinib resistance [80].

*MiR-320a.* miR-320 acts as a tumor suppressor by targeting genes involved in Wnt and IGF pathways. In imatinib resistant GIST patients, down-regulation of the tumor suppressor miR-320a is associated with direct up-regulation of β-catenin and subsequent enhanced expression of anti-apoptotic MCL1 [81].

*MiR-218.* According the findings of a study by Fan and colleagues, the expression of miR-218 was down-regulated in an imatinib-resistant GIST cell line, whereas miR-218 overexpression was able to restore the sensitivity of GIST cells to imatinib, with the PI3K/AKT signaling pathway possibly involved in the mechanism. The PI3K/AKT pathway is downstream of KIT and it is reactivated when GISTs become resistant to imatinib [82].

*MiR-518a-5p.* By performing a microarray analysis on GIST samples from patients resistant to imatinib, miR-518a-5p was found to be a potential predictor of drug sensitivity. Downregulation of miR-518a-5p is likely to upregulate PIK3C2A, causing resistance to imatinib in GISTs. PIK3C2A belongs to the phosphoinositide 3-kinase (PI3K) family, whose member proteins have roles in signaling pathways involved in cell proliferation, oncogenic transformation, cell survival, cell migration and intracellular protein trafficking [83].

*MiR-92a-3p, miR-99a-5p, and miR-101-3p.* MiR-92a-3p, miR-99a-5p and miR-101-3p are three miRNAs implicated in cell cycle regulation. MiR-92a-3p targets Cyclin-dependent kinase inhibitor 1C (CDKN1C), miR-92a-3p regulates mTOR pathway [84], and miR-101-3p also acts as a regulator of the mTOR pathway by mediating AKT activation [85]. The findings of a study, which analyzed miRNA expression profiles across a series of imatinib resistant and sensitive FFPE GIST samples, identified the previously mentioned miRNAs as differentially expressed and therefore possibly implicated in imatinib resistance [86].

*MiR-28-5p.* A recent study identified miR-28-5p as a potential mediator of imatinib resistance in GISTs. Being overexpressed in imatinib-resistant GIST samples and displaying a significant correlation to imatinib response, miR-28-5p has been proven to function as an oncomiR. However, very little is known about this miRNA and therefore further research is required to confirm these findings [87].

### 6.2. Sorafenib

Sorafenib is a small molecule which inhibits multiple kinases involved in tumor cell signaling, proliferation, angiogenesis and apoptosis. Sorafenib is currently in clinical use for unresectable HCC. Its approval was based on the successful outcome of the pivotal SHARP and Sorafenib Asia-Pacific trials in Child-Pugh class A patients with advanced HCC. However, only approximately 30% of patients clinically benefit from sorafenib, and this subgroup usually acquires drug resistance within 6 months. Recent studies have highlighted the role of epigenetics, transport processes, regulated cell death, and the tumor microenvironment in the initiation and development of sorafenib resistance in HCC [88].

*MiR-122.* MiR-122 is the most abundant liver-specific miRNA and it is significantly down-regulated in HCC. miR-122 acts as a tumor suppressor in the liver by inhibiting survival of cancer cells, anchorage-independent growth, migration, invasion, and EMT. The in vitro restoration of expression of miR-122 has been found to sensitize HCC cells to sorafenib. MiR-122 is a negative regulator of A Disintegrin and metalloproteinase domain-containing protein 10 (ADAM10) and serum response factor (SRF), which are both involved in EMT (SFR is also implicated in tumor angiogenesis) and insulin growth factor 1 receptor (IGF1R), which activates the downstream RAS/RAF/ERK pathway, induces proliferation and promotes metastasis [89].

*MiR-34.* In a study by Yang and colleagues, miR-34 was reported to be downregulated in FFPE HCC samples from patients and HCC cell lines and was associated with poorer survival. MiR-34, which is a direct target of p53, binds the 3′-UTR region of the anti-apoptotic protein B-cell lymphoma 2 (Bcl-2), which was found to be overexpressed in HCC samples and the cells analyzed. The restoration of miR-34a potentiated sorafenib-induced apoptosis, suggesting that miR-34a enhanced the anti-tumor effect of sorafenib in HCC cells [90].

*Let-7.* To assess the role of miRNAs in HCC, Ohta et al. performed microarray analysis and discovered that let-7 as was downregulated in human HCC cells. Furthermore, it was also found that the upregulation of let-7 was linked to a decreased expression of its putative target, the anti-apoptotic protein B-cell lymphoma xL (Bcl-xL). Ultimately, the expression of let-7c enhanced apoptosis of HCC cells upon exposure to sorafenib, which is responsible for the downregulation of another anti-apoptotic Bcl-2 protein, MCL1. To summarize, let-7 exerts a tumor suppressing function by inducing apoptosis of HCC cells [91].

*MiR-338-3p.* Hypoxia being one of the main contributors to anti-tumor drug resistance in solid malignancies, hypoxia-inducible factor 1 (HIF-1) has been recognized as one of the key mediators of resistance to sorafenib in HCC. In a study on both patient samples and HCC cell lines, it was discovered that miR-338-3p directly targeted HIF-1α and downregulated the expression of HIF-1α target genes involved in the hypoxia-induced signaling pathway. Via this mechanism, miR-338-3p inhibits HCC tumor growth and sensitizes HCC cells to sorafenib [92].

*MiR-216a/217.* According to the findings of a study by Xia and colleagues on a series of HCC FFPE samples, chemoresistance against sorafenib is caused by overexpression of the miR-216a/217 cluster. Upregulation of miR-216a/217 is able to induce EMT of cancer cells and decrease expression of SMAD7 and PTEN, consequently activating the transforming growth factor β (TGF-β) and PI3K/AKT pathways [93].

*MiR-93.* Following a comprehensive miRNA expression profiling using HCC cell lines, miR-93 was identified as a novel target associated with HCC. MiR-93 acts by binding with the 3′-UTR of PTEN and cyclin-dependent kinase inhibitor (CDK1NA), inhibiting their expression and, as a result, activating the oncogenic PI3K/AKT pathway. In light of these findings, miR-93 expression was also found to render HCC cells more sensitive to sorafenib [94].

*MiR-21.* As previously mentioned, miR-21 dysregulates PTEN, by inhibiting Akt activation. The Akt signaling pathway appears to be highly activated in sorafenib-resistant HCC cells. Moreover, autophagy seems to promote sorafenib sensitivity in sorafenib-resistant HCC cells. Compelling evidence suggests that miR-21 plays a key role in mediating resistance to sorafenib. For example, exposure of HCC cell lines to sorafenib led to an upregulation of miR-21 and a downregulation of PTEN. Furthermore, transfection of miR-21 in HCC cells was able to restore sorafenib resistance by inhibiting autophagy. To summarize, miR-21 contributes to acquired resistance to sorafenib by suppressing autophagy by modulating the Akt/PTEN pathway [95].

*MiR-193a.* MiR-193a acts a tumor suppressor by negatively regulating the pro-metastatic factor urokinase-type plasminogen activator (uPA) and is downregulated in HCC. Transfection of HCC cell lines with miR-193a was found to decrease proliferation, promote apoptosis and enhance sorafenib anti-tumor activity [96].

*MiR-193b.* Substantial downregulation of miR-193b and overexpression of its target MCL1 were observed in HBV-positive HCC cells. MCL1 is an anti-apoptotic protein, which is overexpressed in many human malignancies, including HCC, and has been known as an important mediator of chemosensitivity in HCC. Restoration of the expression of miR-193b sensitized HBV-positive HCC cell lines to sorafenib by facilitating sorafenib-induced apoptosis [97]. Similarly, in another study, HCV-positive HCC cells showed decreased expression of miR-193b and upregulation of its target MCL1 [98].

*MiR-221.* The results of a study by Fornari and colleagues indicated that in both HCC cell lines and xenografts, miR-221 overexpression was linked with sorafenib resistance, by targeting caspase-3 and thereby exerting an anti-apoptotic and oncogenic function. Moreover, when investigating the putative role of miR-221 as a circulating biomarker in HCC patients, lower pre-treatment circulating miR-221 levels were detected in sorafenib responders [99].

*MiR-486.* In HCC tissues and cell lines, miR-486 appears to function as a tumor suppressor by targeting *CITRON* and *CLDN1*, two genes which are responsible for the regulation of cell proliferation and invasion. In addition to being downregulated in HCC, miR-486 was found to enhance chemosensitivity of HCC cells to sorafenib [100].

*MiR-494.* In a recent study by Pollutri et al., miR-494 overexpression was reported to enhance sorafenib resistance via mTOR pathway activation both in vitro and in vivo. In fact, p27, PTEN and p53-upregulated-modulator-of-apoptosis (PUMA) were identified as targets of miR-494, contributing to speed up cell cycle progression, survival and invasiveness. Moreover, high miR-494 expression seemed to be associated with stem-like features [101].

*MiR-101.* As part of the tumor microenvironment, HCC-associated macrophages accelerate tumor progression by releasing growth factors. High TGF-β expression in M2 polarized macrophages is thought to increase tumor growth, metastases and EMT. In HCC cell lines, it was observed that miR-101 targeted dual specificity phosphatase 1 (DUSP1), inhibited TGF-β activation and enhanced the effect of sorafenib in HCC cells by potentiating macrophage modulation of the innate immune responses [102].

### 6.3. Regorafenib

Regorafenib is small-molecule multiple kinase inhibitor. In mCRC, it is indicated for patients who have been previously treated with, or are not considered candidates for, available therapies, including chemotherapy, an anti-VEGF therapy and, if RAS wild-type, an anti-EGFR therapy [103]. It is also licensed as second-line therapy for advanced HCC patients [104] and as third-line therapy for metastatic or unresectable GIST patients [105].

Despite the observed survival benefits, resistance to regorafenib is fairly common. Moreover, toxicity is not insignificant and the absolute clinical benefit is rather small. Therefore, it is crucial to identify efficient predictive biomarkers in order to optimize the use of regorafenib [106].

*MiR-34a.* In a recent study on CRC cell lines, Cai et al. showed that regorafenib is able to reduce stemness and tumorigenesis in vitro, by upregulating the tumor suppressor miR-34, which targets the WNT/β-catenin pathway. Thereby it can be inferred that regorafenib is able to suppress the generation of drug-resistant cancer stem-like cells via modulation of miR-34a-associated signaling [107].

*MiR-30a-5p.* Signal transducer and activator of transcription 3 (STAT3) is a transcriptional factor which contributes to drug resistance in cancer therapies by promoting tumor growth and cancer stemness. miR-30a-5p is a STAT3 downstream miRNA and targets Heat Shock Protein Family A Member 5 (HSPA5), which is a master regulator of unfolded protein response (UPR). A dysregulation of the STAT3-miR-30a-5p-HSPA5 axis, with a downregulation of miR-30a-5p, was observed in regorafenib-resistant in CRC tumorspheres [108].

In the context of liquid biopsies, a few preliminary studies have been carried out to investigate putative circulating predictive biomarkers. In a recent study by Schirripa and colleagues, an miRNA signature involving c-miR-21, c-miR-221 and c-miR-760 was found to be prognostic and predictive of a response to regorafenib in an exploratory cohort of CRC patients. However, the results were not confirmed by the validation cohort [103]. In another study on a large cohort of HCC patients, of the 750 miRNAs analyzed, increased plasma levels of miR-30a, miR-122, miR-125b, miR-200a, and miR-374b decreased levels of miR-15b, miR-107 and miR-320b, and absence of miR-645 were all predictive of survival benefit with regorafenib [109].

## 7. Immune Checkpoint Inhibitors

The introduction of immune-checkpoint blockades in precision oncology led to a paradigm change in the management of advanced cancers. The rationale behind these novel therapies is that cancer cells are able to evade immunosurveillance through different mechanisms, including activation of immune checkpoint pathways that suppress antitumor immune responses [110].

Immunotherapy has recently been incorporated in treatment regimens of GI malignancies. The immune-checkpoint inhibitors currently in clinical use in GI oncology are nivolumab and pembrolizumab, which target programmed death-1 (PD-1) and ipilimumab, which targets cytotoxic T-lymphocyte antigen-4 (CTLA-4) [111].

Immunotherapy approaches have been extensively studied in CRC. In the metastatic setting, ICI therapies provide clinical benefit to defective mismatch repair and microsatellite instable (dMMR/MSI) tumors. Pembrolizumab has been approved as first-line treatment in dMMR/MSI mCRCs based on the successful outcomes of the KEYNOTE-177 trial [112].

As with gastroesophageal cancers, pembrolizumab is currently licensed for recurrent locally advanced or metastatic cancers with PD-L1 expression [113]. Much like in CRC, MSI gastric cancers greatly benefit from ICIs [114]. Moreover, EBV-positive GCs have shown an even more promising response to pembrolizumab [115]. ICIs have also been recently approved as second-line therapy in HCC, after a single agent checkpoint blockade trials obtained partial success [116].

However, a subset of patients who initially responded to ICIs, later relapsed and acquired therapeutic resistance [117]. The role of miRNAs as predictors of response to ICIs has not been elucidated yet. However, several studies have explored how miRNAs regulate the immune checkpoint signaling pathways, leading the way for the discovery of new predictive biomarkers.

Helicobacter Pylori, which is the most common cause of GC, promotes PD-L1 expression and causes an immune escape by downregulating miR-200b and miR-152. Furthermore, a single nucleotide mutation in 3′-UTR of PD-L1 leads to protein overexpression by disrupting the complementarity between miR-570 and its 3′-UTR binding site. This mutation is linked with high PD-L1 levels in GCs [118]. Moreover, according to the findings of a large population-based study from The Cancer Genome Atlas (TGCA) project, a cluster of EBV-miRNAs is linked with a high expression of PD-1/PD-L1 in solid malignancies, including GC [115]. In the context of hematological malignancies, recent studies have found that EBV-associated lymphomas showed high levels of PD-L1. By restoring the expression of PD-L1 targeting tumor suppressor miRNA miR-34a in vitro, PD-L1 expression was reduced and the tumor immunogenicity was increased [119,120]. Thereby, it could be inferred that miR-34a overexpression could be used to subvert PD-L1 induction in EBV-associated neoplasms. Moreover, miR-34a has been found to be frequently methylated in GIST [121].

In CRC, the tumor suppressor miR-138-5p acts by downregulating PD-L1, leading to cancer cell growth in vitro and tumorigenesis in vivo [122]. In a recent study on a series of CRC samples, low miR-200 and high *ZEB* oncogene expression, which is a profile compatible with EMT, was associated with upregulation of PD-L1 [123]. Moreover, a comprehensive miRNA screening using the TCGA dataset led to the identification of miR-148a-3p as a potential negative regulator of PD-L1 expression, particularly in dMMR/MSI CRC [124].

**Figure 2:**
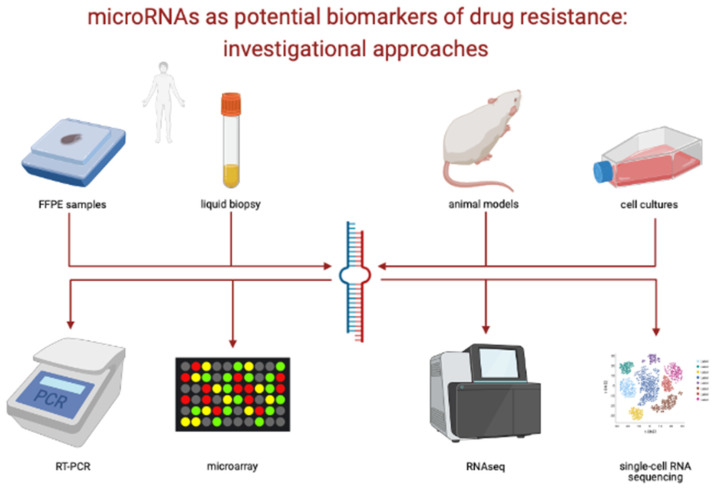
Investigational approaches of miRNAs as potential predictive biomarkers. Credits to BioRender.com, accessed date 18 March 2021.

MiRNAs involved in drug resistance to targeted therapies in GI cancers are summarized in Table 1. The biological materials and molecular techniques used to investigate miRNAs as predictive biomarkers are outlined in Figure 2.

## 8. Conclusions

MicroRNAs are well-documented post-transcriptional regulators of gene-expression in physiological and pathological conditions. They are expressed in an organ- and tissue-specific manner and function through different molecular mechanisms. Given that miRNAs can modulate several oncogenic and tumor-suppressing pathways, increasing evidence points out their role as mediators of drug resistance and as therapeutic options. Because miRNAs can be detected rapidly and efficiently in tissues and biofluids, they are the ideal candidate biomarkers to identify patients with primary resistance to a specific targeted therapy and those who have developed acquired resistance. However, regardless of the numerous studies on the matter, only some miRNAs have been extensively proven to act as mediators of resistance to targeted therapies in GI malignancies, by using large patient cohorts for discovery and validation, and in vitro and in vivo modeling for confirmation. In the era of personalized medicine, more investigational studies are needed to translate the use of miRNA to monitor and forecast treatment response and resistance in a clinical setting.

## Figures and Tables

**Table 1:** miRNAs involved in resistance to targeted therapies in GI cancers.

Targeted Therapies Class	Type of Cancer	MiRNAs Involved	Gene Mechanism Involved	Action
Anti-EGFR	mCRC	Let-7	KRAS downregulation activity	1. high levels were significantly associated with better survival outcome in patients with KRAS mutation [31,32]
		miR-31-5p/miR-31-3p	BRAF activation and role in the signaling pathway downstream of EGFR	2. high levels are associated with TTP and shorter PFS in wt RAS patients treated with cetuximab [33,34,35]
		miR-10/miR-125b	Increasing of Wnt signaling	3. high levels are associated with cetuximab resistance [36]
		miR181a	Inibition of WIF-1	4. high levels are correlated with a longer PFS [37]
		miR-345	EGFR pathway dysregulation	5. high levels are associated with lack of response to cetuximab and irinotecan [38]
		miR-199/miR-375	PHLPP1 and downregulation of Akt pathway	6. high levels correlate with cetuximab resistance [39]
		miR-302	Downregulation of NFIB/ITGA6 axis	7. upregulation restored cetuximab responsiveness [40]
		miR 141-3p	EGFR downstream pathway	8. upregulation improves cetuximab activity [41]
Anti-VEGF	mCRC	miR-126	Enhancing the angiogenic effect of VEGF	9. high levels are associated with resistance to bevacizumab [50]
		miR-664-3p/miR-455-5p	Downregulation of the neuroligin and VRGF system	10. potential predictive tissue biomarkers of bevacizumab effectiveness [51]
		miR-20b-5p/miR-29b-3p/miR-155-5p	Inhibition of Akt pathway/Controlling of HIF-1α signalling	11. high levels are associated with a better outcome of mCRC patients treated with a bevacizumab and chemotherapy [52,53,54]
Anti-HER2	GC	miR-21	PTEN deregulation	12. high levels result in decreased sensitivity to trastuzumab [64]
		miR-223, miR-125b	FBXW7 decrease/PPP1CA downregulation	13. high levels reduce the sensitivity to trastuzumab [67,71]
		miR-16	Akt downregulation via FUBP1 action	14. overexpression is predictors of trastuzumab sensitivity [66]
		miR-200c	EMT block by ZEB1 and ZEB2 targeting	15. downregulated in trastuzumab-resistant GC [70,71]
	GIST	miR-494	FGFR2 reduced expression	16. restores lapatinib sensitivity [75]
RTKs inhibitors	GIST	miR-125a-5p	PTPN18 regulation	17. modulates imatinib response [80]
		miR-320a, miR-518a-5p	Enhanced MCL1 expression via B-catenin/PIK3C2A upregulation	18. downregulated in imatinib-resistant GIST [80,83]
		miR-218	Inhibition of PI3K/AKT pathway	19. overexpression is able to restore the sensitivity to imatinib [82]
		miR-28-5p	NA	20. overexpressed in imatinib resistant GIST samples [87]
	HCC	miR-122, miR-34, let-7, miR-338-3p, miR-93, miR-193a/b, miR-486, miR-101	Downregulation of ADAM10/SRF/Bcl2/Bcl-xL/HIF-1α/CIT-RON/CLDN1/DUSP1	21. overexpression sensitizes HCC cells to sorafenib [89,90,91,92,94,96,97,98,100,102]
	HCC	miR-216a/217, miR-21, miR-221, miR-494	TGF-β and PI3K/AKT pathways activation/inhibition of Caspase 3/mTOR activation	22. overexpression causes chemoresistance against sorafenib [93,95,99,101]
	mCRC	miR-34a	WNT/β-catenin pathway downregulation	23. overexpression sensitizes CRC cells to regorafenib [107]
		miR-30a-5p	Dysregulation of STAT3-HSPA5 axis	24. downregulation is observed in regorafenib-resistant CRC [108]
Immune checkpoint inhibitors	GC	miR-200b, miR-152, miR-570		25. downregulation promotes PD-L1 expression [118]
	mCRC	miR-138-5p, miR-148a-3p		26. overexpression acts by downregulating PD-L1 [122,124]
		miR-200		27. downregulation promotes PD-L1 expression [113]

Abbreviation: mCRC, metastatic colorectal cancer; GC, gastric cancer; GIST, gastrointestinal stroma tumor; TTP, time to progress PFS, progression-free survival; HCC, hepatocellular carcinoma.

## Data Availability

Data available upon request.
